# Microbial Production of Biodegradable Lactate-Based Polymers and Oligomeric Building Blocks From Renewable and Waste Resources

**DOI:** 10.3389/fbioe.2020.618077

**Published:** 2021-02-04

**Authors:** John Masani Nduko, Seiichi Taguchi

**Affiliations:** ^1^Department of Dairy and Food Science and Technology, Faculty of Agriculture, Egerton University, Egerton, Kenya; ^2^Department of Chemistry for Life Sciences and Agriculture, Faculty of Life Sciences and Agriculture, Tokyo University of Agriculture, Tokyo, Japan

**Keywords:** bioplastics, LA-based polymers, LA-based oligomer, lignocellulosic biomass, microbial secretion, PLA

## Abstract

Polyhydroxyalkanoates (PHAs) are naturally occurring biopolymers produced by microorganisms. PHAs have become attractive research biomaterials in the past few decades owing to their extensive potential industrial applications, especially as sustainable alternatives to the fossil fuel feedstock-derived products such as plastics. Among the biopolymers are the bioplastics and oligomers produced from the fermentation of renewable plant biomass. Bioplastics are intracellularly accumulated by microorganisms as carbon and energy reserves. The bioplastics, however, can also be produced through a biochemistry process that combines fermentative secretory production of monomers and/or oligomers and chemical synthesis to generate a repertoire of biopolymers. PHAs are particularly biodegradable and biocompatible, making them a part of today’s commercial polymer industry. Their physicochemical properties that are similar to those of petrochemical-based plastics render them potential renewable plastic replacements. The design of efficient tractable processes using renewable biomass holds key to enhance their usage and adoption. In 2008, a lactate-polymerizing enzyme was developed to create new category of polyester, lactic acid (LA)–based polymer and related polymers. This review aims to introduce different strategies including metabolic and enzyme engineering to produce LA-based biopolymers and related oligomers that can act as precursors for catalytic synthesis of polylactic acid. As the cost of PHA production is prohibitive, the review emphasizes attempts to use the inexpensive plant biomass as substrates for LA-based polymer and oligomer production. Future prospects and challenges in LA-based polymer and oligomer production are also highlighted.

## Introduction

Conventional plastics derived from fossil fuels have become indispensable in modern human life owing to their superior chemical and physical properties that give them versatile qualities such as lightness, robustness, durability, and resistance to degradation (Możejko-Ciesielska and Kiewisz, 2016; Narancic and O’Connor, 2019). Plastics have effectively replaced a number of materials/substances and have found broad applications in industrial, domestic, and medical fields as disposable gears, packaging, furniture, machinery frames, and other accessories (Anjum et al., 2016; Narancic and O’Connor, 2019). Global plastic production reached 359 million tons in 2018 with projected increase in production (Plastics Europe, 2019). The production of plastics has raised environmental concerns because plastics are non-biodegradable and persistent, and they accumulate in the environment, posing threat to life on earth (Alcântara et al., 2020). Although plastics can be recycled, only a small percentage of the plastics (6% of total plastic demand) are recycled because recycling is a time-consuming process, and it also alters the properties of plastic materials, resulting in low-quality plastics (Narancic and O’Connor, 2019). Most of the plastics are disposed in landfills where their degradation is low or used for energy recovery. Disposal of plastics by incineration generates toxic by-products such as furans, polychlorinated biphenyls, dioxins, and mercury and is expensive (Verma et al., 2016; Raza et al., 2018).

**FIGURE 1:**
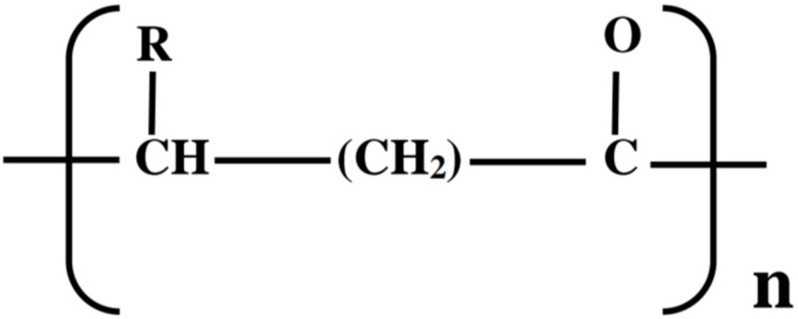
Chemical structure of PHAs; *R* = methyl (C1) to tridecyl (C13).

As a result of the challenges and concerns with petroleum-derived plastics along with the paradigm shift toward development of bioeconomies, researchers have been compelled to explore new approaches of synthesizing biologically produced plastics (bioplastics) that are biodegradable, eco-friendly, and produced from plant biomass/renewable resources as plastic alternatives (Nduko et al., 2015; Możejko-Ciesielska and Kiewisz, 2016; Alcântara et al., 2020). Such bioplastics include the polyesters, polyhydroxyalkanoates (PHAs) and polylactic acid (PLA), which possess similar physicochemical, mechanical, and thermal properties as conventional plastics (Taguchi, 2010; Nduko et al., 2012a; Kourmentza et al., 2017). PHAs belong to a class of natural biodegradable and biocompatible polyesters that are accumulated under unbalanced growth conditions as intracellular carbon and energy reserves by various microorganisms (Możejko-Ciesielska and Kiewisz, 2016; Tarrahi et al., 2020). Currently, more than 150 different 3-, 4-, 5-, and 6-hydroxy fatty acid monomer constituents (Figure 1) containing straight, branched, saturated, unsaturated, and aromatic structures (*R*-side chain) in PHA have been documented in more than 90 genera of microbial species (Anjum et al., 2016; Meng and Chen, 2018; Raza et al., 2018). Based on carbon chain length of the monomeric unit (*R*), PHAs have been classified into three classes (Carpine et al., 2020);

i Short-chain-length PHAs (scl-PHAs). The monomeric units consist three to five carbon atoms such as polyhydroxybutyrate [P(3HB)], poly(4-hydroxybutyrate) P(4HB), and poly(3-hydroxyvalerate) P(3HV; Anjum et al., 2016). These polymers are produced by organisms such as *Cupriavidus necator* and *Alcaligenes latus* (Lee and Na, 2013; Raza et al., 2018). These scl-PHAs exhibit thermoplastic material properties similar to polypropylene (PP; Wang et al., 2016).
ii Medium-chain-length PHAs (mcl-PHAs). Their monomeric units consist 6 to 14 carbon atoms. They are produced by several bacteria such as *Pseudomonas putida* and *Pseudomonas mendocina*. The mcl-PHAs are flexible and elastic similar to rubber. They also possess low crystallinity and tensile strength and have high melting point and glass-transition temperatures (Nduko et al., 2012a; Gopi et al., 2018).
iii Long-chain-length PHAs. Their monomeric units have carbon atoms more than 14. These have been documented in the bacteria *Shewanella oneidensis* and *Aureispira marina* (Suriyamongkol et al., 2007).

Among the PHAs, P(3HB) was the first to be identified in 1926 in the bacterium *Bacillus megaterium* (Lemoigne, 1926). P(3HB) has been widely studied and characterized, and cells accumulate P(3HB) up to 92% of the cell dry weight (Norhafini et al., 2019). This polymer has similar thermal properties to that of PP., However, P(3HB) has difficulties in processability because of its brittle nature, limiting its applications (Kourmentza et al., 2017). As a result, P(3HB) has been copolymerized with a number of monomers such as 3-hydroxyvalerate (3HV) to form a copolymer, P(3HB-*co*-3HV), which has improved material properties (tougher and flexible and acquires broader thermal processing properties) compared with P(3HB) homopolymer (Grigore et al., 2019). Copolymerization of 3HB with 3-hydroxyhexanoate (3HHx) generated a copolymer, P(3HB-*co*-3HHx), which is produced at industrial scale by Kaneka Co. Ltd (López et al., 2015; Nduko and Taguchi, 2019).

Polylactic acid is one of the most promising biodegradable and biocompatible aliphatic thermoplastic with extensive mechanical property profile (Li et al., 2020). PLA is obtained from lactic acid (LA), a naturally occurring organic acid produced by microbial fermentation from renewable resources such as sugars obtained from corn, cane sugar, and sugar beets (Van Wouwe et al., 2016). On hydrolysis, PLA produces LA; hence, PLA can be produced and used as an environment-friendly material (Singhvi et al., 2019). PLA is highly versatile, high-strength, and high-modulus polymer that yields materials with potential use in industrial packaging or as biocompatible medical devices (Matsumoto and Taguchi, 2010). PLA has good processability, and its manufacturing is amenable and can adapt the conventional plastic equipment for production of molded parts, films, and fibers (Castro-Aguirre et al., 2016). LA is a chiral molecule existing in both L and D isomers. These monomers can be polymerized into pure poly-L-LA (PLLA), pure poly-D-LA (PDLA), or poly-D-LLA, giving high-molecular-weight crystalline, or amorphous polymers (Auras et al., 2004; Tyler et al., 2016).

Polylactic acid is normally synthesized in three steps: (i) LA production by microbial fermentation, (ii) LA purification followed by cyclic dimer formation (lactide), and (iii) polycondensation of LA or ring-opening polymerization (ROP) of the cyclic lactides (Auras et al., 2004; Castro-Aguirre et al., 2016). Polycondensation is the least expensive route for PLA synthesis; however, it does not give a solvent-free high-molecular-weight PLA (Auras et al., 2004). Consequently, ROP has emerged as the most common route to synthesize high-molecular-weight PLA (Pretula et al., 2016). ROP consists of three processes: polycondensation, depolymerization, and the ring opening of the LA cyclic dimer in the presence of a catalyst (Auras et al., 2004). The process also involves complicated and expensive purification steps, rendering the ultimate products more expensive compared to their petroleum-based counterparts (Taguchi, 2010). The catalysts used are mostly transition heavy metals, particularly tin, aluminum, lead, zinc, bismuth, ion, and yttrium (Mehta et al., 2005). However, trace residues of the heavy metal catalysts are unwanted in materials for specific applications such us in medical and food contact surfaces (Taguchi, 2010; Nduko et al., 2012a; Matsumoto and Taguchi, 2013a). Therefore, the replacement of heavy metal catalysts with safe and environmentally acceptable alternatives has been important concern.

Enzymatic polymerization of LA monomers has emerged as one of the most viable and environmentally benign alternative methods for PLA production (Riaz et al., 2018). In the year 2008, Taguchi et al. (2008) established for the first time a whole-cell biosynthetic system. In that system, LA was polymerized alongside 3HB to produce P(LA-*co*-3HB) copolymer without the need for heavy metal catalysts, extremely pure monomers, anhydrous conditions, and high temperatures. From these pioneering studies, efforts have been made toward the synthesis of P(LA-*co*-3HB), PLA homopolymer, and polymerizing other monomers such as glycolate and 2HB using microbes (Matsumoto et al., 2011a, 2013; Yang et al., 2013; Nduko and Taguchi, 2019). In addition, a system for the production of LA oligomers by microorganisms has been established (Utsunomia et al., 2017c). These oligomers are polymerized into P(LA-*co*-3HB), thereby shortening the process for PLA synthesis.

Polyhydroxyalkanoates and PLA have found many applications in domestic, agricultural, and industrial sectors but mostly in the medical field (Albuquerque and Malafaia, 2018). The polymers have been produced and improved for diverse biomedical applications such as making absorbable sutures, nerve guides, bone marrow scaffolds, repair patches, cardiovascular patches, heart valves, nerve repair devices, tendon repair devices, orthopedic pins, tissue engineered cardiovascular devices, articular cartilage repair devices, guided tissue repair/regeneration devices, and wound dressings (Masood et al., 2015; Elmowafy et al., 2019). The PHAs and PLA have also been used as nanoparticles for drug delivery, biocompatible porous implants and implant coatings, scaffolds, and tissue engineering applications such as antibacterial agents (Gadgil et al., 2017). PHAs also have potential use in the formation of necessities such as packaging materials, consumer and household goods, furniture, sports, health and safety automotive and transport sector, and the construction and building sector (Vijayendra and Shamala, 2014; Elmowafy et al., 2019). This indicates that PHAs and PLA have high potential, and their optimum production is of interest.

In this review, we highlight novel ideas that introduce concepts of the microbial production of P(LA-*co*-3HB), PLA, and LA oligomers using engineered microbes. We discuss the creation of the microbial cell factory for P(LA-*co*-3HB) synthesis and give a synopsis of LA monomer improvement in the copolymer and eventual PLA biosynthesis, properties and degradation of the LA-based polymers [more information was discussed in Nduko et al. (2015)], the use of integrated lignocellulosic biomass refineries for polymer and oligomer production, and the recent reports on the enabling technologies such as metabolic engineering, protein engineering and genetic engineering, and fermentation media manipulation strategies for the production of the renewable polymers. This article also gives a snapshot on the challenges of LA-based polymer production and future perspectives on their production.

## Biodegradable Biopolyesters: PHAs and PLA

Among the well-studied biopolyesters are PHAs and PLA. These polyesters are biodegradable and have thermoprocessibility and flexible mechanical properties similar to conventional plastics such as PP and low-density polyethylene (Castro-Aguirre et al., 2016; Shi et al., 2020).

### Polyhydroxyalkanoates

Polyhydroxyalkanoates are a large family of biopolyesters produced by many bacteria for carbon and energy storage (Możejko-Ciesielska and Kiewisz, 2016). Polyhydroxybutyrate (PHB) was the first to be discovered and is the most studied PHA member (Anjum et al., 2016). More than 150 hydroxyalkanoic acid monomer structures have been reported in PHAs (Raza et al., 2018; Sagong et al., 2018; Surendran et al., 2020). The diversity of monomeric structures gives flexible properties of PHAs such as brittleness, elasticity, and stickiness (Raza et al., 2018). PHAs can be produced by either the wild-type, pure/mixed bacteria or by metabolically engineered bacteria including *Ralstonia eutropha*, *Wautersia eutropha*, *Corynebacterium glutamicum*, *Azotobacter* sp., *Bacillus* sp., *Pseudomonas* sp., *Burkholderia* sp., *Halomonas* sp., *Aeromonas* sp., *Rhodobacter sphaeroides*, and *Escherichia coli*, among others (Song et al., 2012; Anjum et al., 2016). PHAs are intracellularly accumulated and can be extracted from cells by organic solvents such as chloroform or by use of alkaline solutions among other methods (Raza et al., 2018). In the recent past, PHA production has been significantly improved through process optimization and metabolic engineering strategies targeting higher yields/productivity and adjustable monomer structures or monomer ratios in the end products (PHAs; Zhang et al., 2020). In addition, system-level analysis has enabled deeper understanding of microbes producing PHA. These studies, along with construction of new PHA production systems, has led to the synthesis of novel PHAs incorporating new monomers, PHAs with variable monomer composition, and regulation of the molecular mass (Koller and Braunegg, 2018).

### Polylactic Acid

**FIGURE 2:**
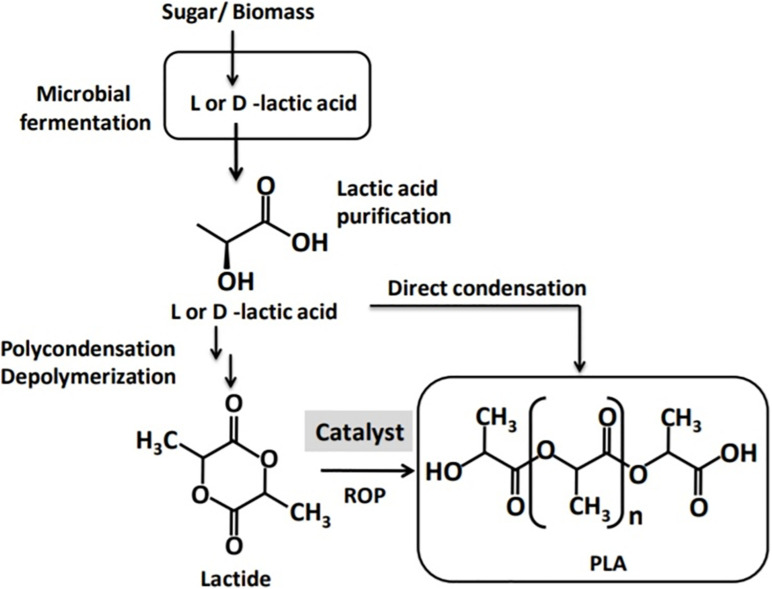
A schematic presentation of polylactic acid (PLA) production from lactic acid by ring-opening polymerization (ROP) and direct condensation.

Polylactic acid is a linear aliphatic polyester produced from renewable carbon sources that possesses desirable properties, making it an attractive alternative to the petroleum-derived plastics (Mehta et al., 2005; Castro-Aguirre et al., 2016). Currently, PLA is produced by solvent-based condensation but more commonly by ROP (Figure 2) through the cyclic intermediate dimer (lactide; Pretula et al., 2016; Singhvi et al., 2019). Condensation produces only low- to intermediate-molecular-weight PLA polymers and is carried out using a solvent and at high vacuum and temperature (Auras et al., 2004). The condensation process for PLA synthesis further requires a large reactor and a number of energy-intensive processes such as evaporation and solvent recovery (Mehta et al., 2005). Because of the challenges with condensation, efforts for PLA production shifted to ROP, and Mitsui Toatsu Chemicals patented an azeotropic distillation process that uses a high-boiling solvent to remove water in a direct esterification process to produce high-molecular-weight PLA. This process was adopted by NatureWorks LLC, a global PLA producer to produce high-molecular weight PLA via ROP for commercial purposes (Castro-Aguirre et al., 2016). With the use of ROP and advances in technology of fermentative LA production, the cost of PLA has come down (Hu et al., 2016). However, compared with petroleum-derived plastic counterparts, the multistep chemo–bio process for PLA production is still complex and expensive, giving PLA a competitive disadvantage (Matsumoto and Taguchi, 2010; Lasprilla et al., 2012; Nduko et al., 2013b; Vink and Davies, 2015; De Clercq et al., 2018). Attempts have been made to alter properties of PLA via blending or copolymerization with other monomers (Matsumoto et al., 2013). Nevertheless, availability of readily polymerizable monomers and their high cost render chemical synthesis of PLA to be unattractive, hence the need for a microbial process for the synthesis of PLA (Singhvi et al., 2019).

## The First Microbial Factory of LA-Based Polymers

**FIGURE 3:**
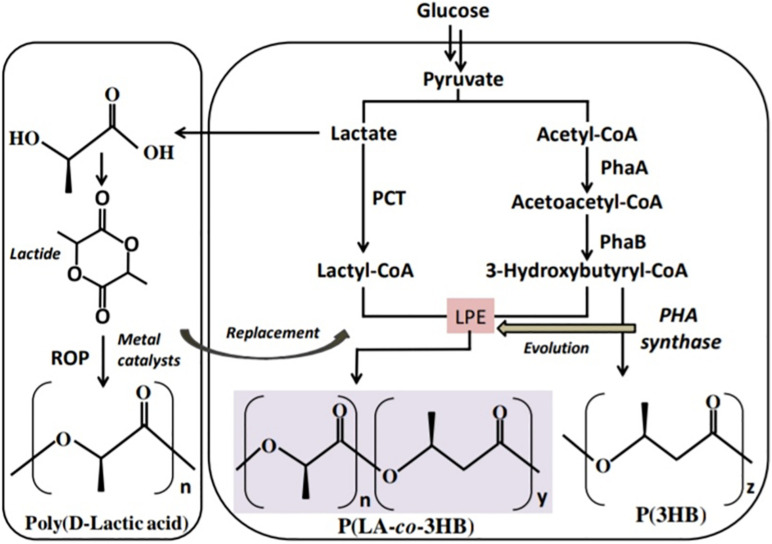
Schematic presentation of the biosynthetic pathway for the production of lactate-based polymers in engineered microorganisms. PCT, propionyl-CoA trabnsferase; PhaA, β-ketothiolase; PhaB, NADPH-dependent acetoacetyl-CoA reductase; LPE, lactate (LA)–polymerizing enzyme.

**FIGURE 4:**
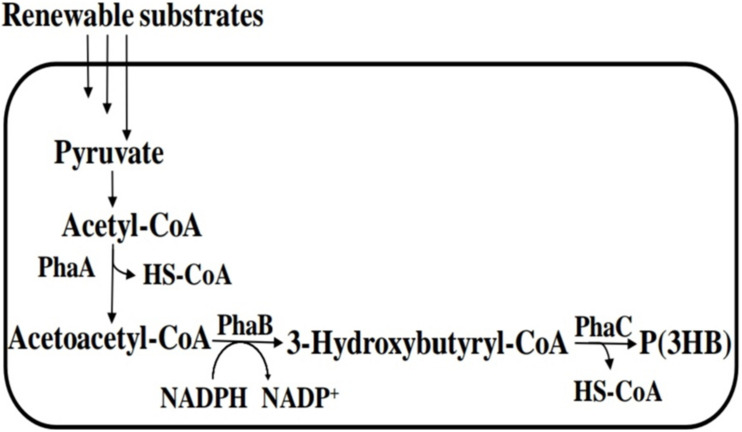
PHA biosynthetic pathway for polyhydroxybutyrate [P(3HB)]. PhaA, β-ketothiolase; PhaB, NADPH-dependent acetoacetyl-CoA reductase; and PhaC, polyhydroxyalkanoate (PHA) syynthase.

The polymerization of LA monomer in a similar version as PHA synthesis was first proposed by Steinbüchel and Valentin (1995). This was based on the similarity between LA and PHA monomers, i.e., the presence of carboxyl and hydroxyl groups. However, by 1995 no LA incorporation in PHAs had been reported (Taguchi et al., 2008). The establishment of a single-step process for the production of PLA in a similar version as PHAs will overcome the use of metal catalysts and purification of LA steps in PLA synthesis and potentially reduce the cost of production (Matsumoto and Taguchi, 2013b; Nduko et al., 2013b; Singhvi et al., 2019). In the year 2008, Taguchi et al. (2008) reported for the first time a whole-cell bioprocess for the production of LA-based PHAs: poly(lactate-*co*-3-hydroxybutyrate) [P(LA-*co*-3HB)] in engineered bacteria (*E. coli*; Figure 3). The system employed the PHA biosynthetic metabolic pathway (Figure 4) using an “LA-polymerizing enzyme (LPE),” replacing the metal catalyst used in the chemical synthesis of PLA as earlier explained (Taguchi et al., 2008). The polymerization of the LA unit was possible after the discovery of a Ser325Thr/Glu481Lys mutant of PHA synthase from *Pseudomonas* sp. 61-3 [PhaC1PS(ST/QK)] that had acquired LA-polymerizing activity and a propionate CoA-transferase (PCT) enzyme that was supplying the monomer, D-lactyl-CoA. This discovery laid the foundation for the advancement of the system by varying monomer ratios and composition (Nduko et al., 2015; Nduko and Taguchi, 2019). Subsequently, a similar procedure using an evolved PHA synthase and PCT has been recruited by other researchers to produce LA-based polyesters in engineered microorganisms (Yang et al., 2013; Choi et al., 2019; Tran and Charles, 2020).

### Production of LA-Based Polymers With Varied LA Fraction

In the first report of LA-based P(LA-*co*-3HB) biosynthesis using glucose as a carbon source, only 6 mol% of LA unit was incorporated into the P(LA-*co*-3HB) copolymer (Taguchi et al., 2008). From literature, it is known that monomer ratio, composition, and distribution in a polymeric molecule affect the mechanical, thermal, optical, and chemical properties of a copolymer because of the changed melting temperatures (*T*_m_) and crystallinity (Menges et al., 2011; Yamada et al., 2011). This was demonstrated by P(6 mol% LA-*co*-94 mol% 3HB) that had lower *T*_m_ compared with P(3HB) and PLA homopolymers (Taguchi et al., 2008). Therefore, monomer ratio, composition, and distribution within a copolymer can be varied to generate new materials (Yamada et al., 2009, 2010; Song et al., 2012; Nduko et al., 2015). In an *in vitro* chemoenzymatic experiment, P(LA-*co*-3HB) containing 36 mol% of LA was produced from equimolar quantities of LA-CoA and 3HB-CoA monomers (Tajima et al., 2009), which was higher than the 6 mol% achieved in an *in vivo* system in engineered *E. coli* JM109 strain (Taguchi et al., 2008). This suggested that the *in vivo* system can be rewired to synthesize LA-based polymers with enhanced LA units or PLA. Following these pioneering studies by Taguchi et al. (2008), various efforts have been made to alter monomer ratio and composition as summarized in Table 1.

**TABLE 1:** History of microbial LA-based polymer production.

Intervention	Achieved results	References
First synthesis of LA-based polymer: P(LA-*co*-3HB)	LA-based polymer synthesis with 6 mol% LA	Taguchi et al., 2008
Use of Δ*pflA* mutant (*Escherichia coli* JW0885) with P(LA-*co*-3HB) biosynthetic pathway	Production of P(LA-*co*-3HB) containing 26 mol% from glucose	Yamada et al., 2009
Use of Δ*pflA* mutant (*E. coli* JW0885) with P(LA-*co*-3HB) biosynthetic pathway under anaerobic conditions	Production of P(LA-*co*-3HB) containing 47 mol% from glucose but with polymer content less than 2% of dry cell weight	Yamada et al., 2009
Use of Δ*pflA* mutant (*E. coli* JW0885) with P(LA-*co*-3HB) biosynthetic pathway having an evolved LPE (eLPE) under aerobic conditions	Production of P(LA-*co*-3HB) containing 47 mol% from glucose with polymer content less than 60% of dry cell weight	Yamada et al., 2010
Use of Δ*pflA* mutant (*E. coli* JW0885) with P(LA-*co*-3HB) biosynthetic pathway having an evolved LPE (eLPE) under anaerobic conditions	Production of P(LA-*co*-3HB) containing 62 mol% from glucose but with polymer content less than 1% of dry cell weight	Yamada et al., 2010
Use of *E. coli* LS5218 expressing evolved LPE on glucose supplemented with valerate under aerobic conditions. P(3HB) biosynthetic pathway was omitted	Production of LA-based polymer containing 90–96 mol% LA units	Shozui et al., 2011
*Corynebacterium glutamicum* Expressing LPE only/and PHB biosynthetic pathway under aerobic conditions on glucose	Production of P(LA-*co*-3HB) containing 97–99.3 mol% LA units	Song et al., 2012
*E. coli* JW0885 (Δ*pflA*) expressing LPE and PHB biosynthetic pathway and cultivated on xylose under aerobic conditions	Production of P(LA-*co*-3HB) containing 34 mol% from xylose	Nduko et al., 2013a
*E. coli* JW0885 (Δ*pflA*) expressing evolved LPE (eLPE) and PHB biosynthetic pathway and cultivated on xylose under aerobic conditions	Production of P(LA-*co*-3HB) containing 60 mol% from xylose	Nduko et al., 2013a
*E. coli* JW2121 (Δ*dld*) expressing evolved LPE (eLPE) and PHB biosynthetic pathway and cultivated on xylose under aerobic conditions	Production of P(LA-*co*-3HB) containing 66 mol% from xylose	Nduko et al., 2014
*E. coli* JWMB1 (Δ*dld*, Δ*pflA*) expressing evolved LPE (eLPE) and PHB biosynthetic pathway and cultivated on xylose under aerobic conditions	Production of P(LA-*co*-3HB) containing 73 mol% from xylose	Nduko et al., 2014
*In vitro* analysis of the P(LA-*co*-3HB) biosynthetic system in *E. coli* and *C. glutamicum*	Production of PLA homopolymer	Matsumoto et al., 2018b
Expression of medium chain length (MCL)–supplying enzymes (PhaG and (*R*)-3-hydroxyacyl-CoA ligase in *E. coli* along with LPE	Production of LA-based polymers with MCL 3HA units with C_3_, C_8_, C_10_, and C_12_ monomers with 92 mol% LA	Goto et al., 2019a
Expression of exogenous of D-lactate dehydrogenase (ldhD) from *Lactobacillus acetotolerans* HT in *E. coli* harboring P(LA-*co*-3HB) biosynthetic genes	Produced P(LA-*co*-3HB) with high LA fractions compared with control cells	Goto et al., 2019b
Expression of PCT and PhaC1400 in *Sinorhizobium meliloti*	Production of PLA homopolymer	Tran and Charles, 2020

Among the strategies employed to enhance LA units in P(LA-*co*-3HB) was the usage of a Δ*pflA* mutant (*E. coli* JW0885; Shozui et al., 2010b). The mutant overproduces LA and its usage as a host expressing the set of enzymes for P(LA-*co*-3HB) synthesis resulted in the production of P(LA-*co*-3HB) with 26.7 mol% LA and a polymer content of 51%. Both the LA fraction and polymer content attained by the *E. coli* JW0885 were higher compared with those of *E. coli* JM109 (6 mol% and 40% for LA and polymer content, respectively). This metabolic engineering strategy was thus effective in enhancing both the LA fraction in the copolymer and the polymer content. The same *E. coli* mutant (*E. coli* JW0885) was cultivated under anaerobic conditions, and this led to the production of P(LA-*co*-3HB) containing 47 mol% LA units (Yamada et al., 2009). This higher LA fraction was, however, at the expense of polymer content as polymer content of the cells was 15%. This called for further interventions to improve the LA fractions in the copolymer without reducing the polymer content in the cells.

In subsequent studies, LPE was further evolved (eLPE) based on beneficial mutations (Chek et al., 2019) and expressed in *E. coli* JW0885 along with the other requisite P(LA-*co*-3HB) synthesis enzymes. This strategy resulted in the production of P(LA-*co*-3HB) having 45 mol% LA units with a polymer content of 62% from glucose (Yamada et al., 2010). When the culture condition was switched to anaerobic, P(LA-*co*-3HB) copolymer with 62 mol% LA unit was produced, however, with polymer content in dry cells being 12% (Yamada et al., 2010). The results demonstrated the power of evolutionary engineering in affecting enzyme specificity and activity.

When P(LA-*co*-3HB) monomer sequence was analyzed by nuclear magnetic resonance (NMR), the LA-LA-LA triad sequence was detected (Yamada et al., 2009). This suggested that LA monomer could link to another LA monomer, implying that an LA-rich P(LA-*co*-3HB) or PLA could be synthesized by microorganisms, although the earlier *in vitro* experiment had indicated that LA polymerization was dependent on the presence of 3HB monomer (Taguchi et al., 2008). Shozui et al. (2011) knocked out PhaA and PhaB (3HB-CoA monomer supplying enzymes) from the P(LA-*co*-3HB) biosynthesis construct to eliminate 3HB supply and instead expressed PhaJ, which could supply 3-HV-CoA from valerate added to the media through the β-oxidation pathway. The *E. coli* JW0885 cells with that system produced P(LA-*co*-3HB-*co*-3HV) with 90–96 mol% of LA units from glucose supplemented with sodium valerate. Although 3HB was incorporated into the formed polymer, this demonstrated that elimination of the 3HB supply pathway was a viable strategy for producing PLA-like polymers.

### Production of LA-Based Polymers in Other Microorganisms

Production of LA-based polymers in engineered *E. coli* was thought to harbor challenges as the organism being a Gram-negative bacterium produces harmful endotoxins such as lipopolysaccharides (Lee et al., 2015; Mamat et al., 2015; Grage et al., 2017). This is challenging especially if the polymers are targeted for food-grade contact surfaces and in biomedical applications (Taguchi et al., 2015). *C. glutamicum*, an endotoxin-free Gram-positive bacterium with GRAS (generally recognized as safe) status that had been applied for the biosynthesis of a number of industrial compounds and PHAs, was selected as host for the production of LA-based polymers (Jo et al., 2006, 2007; Taguchi et al., 2015). Consequently, the requisite P(LA-*co*-3HB) synthesis pathway was assembled in *C. glutamicum* and the host cultivated for polymer production from glucose. In stark contrast with *E. coli* (45 mol% of LA, 62% polymer content), the P(LA-*co*-3HB) produced in *C. glutamicum* had 97 mol% of LA fraction and 2.4% polymer content with the same set of genes (Song et al., 2012). The high LA fraction in *C. glutamicum* was attributed to the low 3HB-CoA supply in *C. glutamicum* as supported by low P(3HB) yield in *C. glutamicum* (1.0%) when PCT (D-LA-CoA supplying enzyme) was omitted from the system. To corroborate this, the researchers omitted PhaA and PhaB (3-HB-CoA supplying enzymes) from the system (Song et al., 2012). Expressing P(LA-*co*-3HB) synthetic system without PhaA and PhaB in *C. glutamicum* resulted in the production of PLA-like polymers [P(99.3 mol%LA-*co*-3HB)] with a polymer content of 1.4%. The detection of 3HB units in the resultant P(LA-*co*-3HB) was attributed to the possible presence of an endogenous 3-HB-CoA supplying pathway in *C. glutamicum*. ^1^H-NMR analysis of P(99.3 mol% LA-*co*-3HB) produced in *C. glutamicum* detected strong resonances of LA that were similar to those of chemically synthesized PLA (Song et al., 2012).

To increase polymer productivity in *C. glutamicum*, Matsumoto et al. (2014) explored the rate-limiting step by quantitative metabolite analysis. Significant quantities of lactyl-CoA were found during polymer synthesis, indicating that rate limitation occurred during polymerization. Subsequently, codon optimization of LPE was conducted to increase its expression level, and this led to a 4.4-fold increase in polymer content compared with the control without significantly affecting lactyl-CoA concentration. In the same study, it was found that acetyl-CoA served as a CoA donor for lactyl-CoA. The study demonstrated the significant of metabolite analysis to improve PLA-like polymer production.

When the polymers produced in *C. glutamicum* and *E. coli* were assessed, it was found that LPE could produce PLA by polymerizing LA-CoA as a sole substrate (Matsumoto et al., 2018b). However, the PLA had low molecular weight that was attributed to the low mobility of the synthesized polymer. It was found that polymerization of LA-CoA and 3-HB-CoA proceeded, dependent on the 3-LA-CoA concentration in the bacterium. For instance, the LA-CoA concentrations in bioengineered *C. glutamicum* were high and detectable, unlike in *E. coli* where LA-CoA concentrations were undetectable. Consequently, where there was low LA-CoA concentration in *E. coli*, P(LA-*co*-3HB) synthesis proceeded more efficiently than in *C. glutamicum* that had high levels of LA-CoA. These results demonstrated the importance of genetic diversity in varying monomer ratios in P(LA-*co*-3HB; Song et al., 2015).

In another study, Tran and Charles (2020) introduced a broad-host-range plasmid (pTAM) that was expressing codon optimized PCT and engineered *Pseudomonas* sp. MBEL 6–19 PHA synthase 1 (PhaC1Ps6-19, PhaC1400) into phaC mutant strains of *Sinorhizobium meliloti* and *P. putida*, which are native PHA producers. *S. meliloti* was found to produce PLA homopolymer with 3.2% polymer content, while *P. putida* produced copolymers containing 3HB, 3HHx, and 3HO with polymer content of up to 42%. These results indicated that exploration of host strains offers another strategy for the production of LA-based polymers and PLA.

### Latest Approaches to LA-Based Polymer Production

To further modulate P(LA-*co*-3HB) yield and monomer composition, other strategies were devised. One such strategy was the disruption of σ factors that govern gene transcription at the global level. Four σ factors, RpoS, RpoN, FliA, and FecI, which are found in *E. coli*, were disrupted and the respective disruptant mutants used for P(LA-*co*-3HB) synthesis (Kadoya et al., 2015a). It was found that Δ*rpoN* mutant had enhanced LA fraction (26.2 mol%) and polymer yield (6.2 g/L) when compared with the parent *E. coli* strain (*E. coli* BW25113; 18.6% mol% and 5.3 g/L for LA fraction and polymer yield, respectively). These beneficial effects in the Δ*rpoN* mutant were attributed to higher LA production in the mutant strain. The Δ*rpo*S mutant on its part produced higher P(LA-*co*-3HB; 5.8 g/L) but had lower LA fraction (12.3 mol%) than that of *E. coli* BW25113 (parent strain; 18.6 mol%). The Δ*rpoN* mutant was grown in xylose that had been demonstrated to give higher LA fraction than glucose for P(LA-*co*-3HB) production (Nduko et al., 2013a, 2014). The P(LA-*co*-3HB) polymer produced with this mutant had 33.9 mol% LA that was higher than 26.2 mol% LA in the polymers produced from glucose by the same mutant (Kadoya et al., 2015b). However, the LA ratios in P(LA-*co*-3HB) produced by Δ*rpoN* mutant cultivated on xylose (33.9 mol%) were similar to those produced by the parent strain (BW25113) cultivated under the same conditions (35.9 mol%), indicating no synergistic effects between *rpoN* disruption and xylose utilization.

Genome-wide transposon mutagenesis of P(LA-*co*-3HB)–producing *E*. *coli* was conducted as another strategy to generate beneficial mutants for P(LA-*co*-3HB) production (Kadoya et al., 2015b). From more than 10,000 mutants generated, a mutant (Δ*mtgA*) was generated that produced significantly higher (5.1 g/L) P(LA-*co*-3HB) than the recombinant parent strain (2.9 g/L). The Δ*mtgA* strain was found to be enlarged in size during P(LA-*co*-3HB) production, and this mutation in *E. coli* BW25113 (JW3175) was evaluated for P(LA-*co*-3HB) production (Kadoya et al., 2015c). The Δ*mtgA* mutation was found to be effective in P(LA-*co*-3HB) accumulation (7.0 g/L) as compared with its parental strain, *E. coli* BW25113 (5.2 g/L), without significant alteration of the LA/3HB ratio. This mutation gave flexibility for expansion during polymer production and thus along with Δ*rpoN* offers an additional strategies for P(LA-*co*-3HB) production.

To enhance LA fraction in P(LA-*co*-3HB), Goto et al. (2019b) introduced exogenous D-lactate dehydrogenase gene (ldhD) from *Lactobacillus acetotolerans* HT into *E. coli* strains expressing P(LA-*co*-3HB) biosynthetic genes. The recombinant strains cultivated on glucose under microaerobic conditions produced P(LA-*co*-3HB) copolymer with LA fractions of 19.8, 15.7, and 28.5 mol% for DH5α, LS5218, and XL1-Blue *E. coli* strains, respectively. These values were higher than those of corresponding strains without the ldhD gene (<6.7 mol% of LA units). This demonstrated ldhD gene expression as an additional strategy to enhance LA-fraction in P(LA-*co*-3HB).

There have been attempts to diversify monomers copolymerizing with LA apart from 3HB. For instance, Shozui et al. (2010a) produced a terpolymer that incorporated up to 7.2 mol% 3HV together with 8.7 mol% LA and 84.2 mol% 3HB. Matsumoto et al. (2013) produced a lactate-based copolymer containing 2HB in *E. coli*. In the study, recombinant *E. coli* LS5218 expressing LPE and PCT were cultivated in a medium containing glucose and 2HB-produced P(86 mol% 2HB-*co*-LA) copolymer. Goto et al. (2019a) produced LA-based polymers containing medium-chain-length 3-hydroxyalkanoates (MCL-3HA) in *E. coli*. The *E. coli* LS5218 strain expressing P(LA-*co*-3HB) biosynthetic genes along with (MCL-3HA) monomer supplying enzymes [(R)-3-hydroxyacyl-ACP thioesterase (PhaG) and (*R*)-3-hydroxyacyl (3HA)-CoA ligase] from the fatty acid biosynthesis pathway produced P(LA-*co*-3HB-*co*-3HA), a copolymer containing 19.7 mol% LA (C_3_), 74.9 mol% 3HB (C_4_), and 5.4 mol% MCL-3HA units of C_8_ and C_10_. When PhaA and PhaB (3HB supplying enzymes) were excluded from the system, P(92.0% LA-*co*-3HA) with an HA containing C_3_, C_8_, C_10_, and C_12_ monomers was produced. This copolymer had transparency similar to that of chemically synthesized PLA (Goto et al., 2019a). Other monomers that have been incorporated along with lactate to form copolymers include glycolate (Choi et al., 2016) and 4HB (Choi et al., 2020).

## Application of Lignocellulosic Biomass for the Production of P(LA-*Co*-3HB)

### Xylose Utilization for Production of P(LA-*co*-3HB)

The substrate to use for P(LA-*co*-3HB) production is important because of the cost implication (substrate can take up to 50% of total production cost; Raza et al., 2018) and its effect on the polymer/copolymer composition (Alcântara et al., 2020). Substrates such as sugars and starch-based substrates are edible and in direct competition with foods and feeds, and thus, the use of the second generation feedstock is essential. This is from the ethical standpoint and the need to prevent increase in food prices and disrupting global food supplies (Koller and Braunegg, 2018). Therefore, there has been efforts by scientists to valorize waste streams as carbon sources for the production of PHAs, which aligns with the biorefinery concept (Al-Battashi et al., 2019). Lignocellulosic biomass is inedible and has demonstrated great potential as a carbon source for PHA production (Matsumoto et al., 2011b; Nduko et al., 2012b; Bhatia et al., 2019; Li and Wilkins, 2020). Lignocellulosic biomass comprises 40–50% cellulose, 25–30% hemicellulose, and 15–20% lignin (Brethauer and Studer, 2015). The cellulose component has been hydrolyzed into glucose and used for the fermentative production of a number of bioproducts. In contrast, the hemicellulose components composed of mainly xylose and the lignin component have not been fully utilized (Pollegioni et al., 2015; Takkellapati et al., 2018; Xu et al., 2020). In a biorefinery where lignocellulosic biomass is utilized, it is essential to fully utilize cellulose and hemicellulose (carbohydrate components) and lignin.

To demonstrate the potential of hemicellulose, Nduko et al. (2013a) evaluated the use of pure xylose for P(LA-*co*-3HB) production. Using *E. coli* JW0885 (*pflA*^–^) transformed with requisite enzymes for P(LA-*co*-3HB) synthesis (LPE along with the 3HB-CoA and LA-CoA monomer supplying enzymes), P(34 mol%LA-*co*-3HB) with 61% polymer content and 5.5 g/L polymer yield was produced from 20 g/L of xylose. This was different from P(26 mol%LA-*co*-3HB) with 62% polymer content and 6.5 g/L polymer yield obtained from 20 g/L of glucose under the same cultivation conditions (Yamada et al., 2009; Nduko et al., 2013a). When eLPE was employed, *E. coli* JW0885 cells cultivated on 20 g/L of xylose produced P(60 mol% LA-*co*-3HB) with a polymer content of 70% and polymer yield of 7.3 g/L, whereas those cultivated in 20 g/L of glucose produced P(47 mol% LA-*co*-3HB) with a polymer content of 81% and polymer yield of 7.9 g/L (Nduko et al., 2013a). Xylose thus demonstrated its advantages in giving P(LA-*co*-3HB) with LA fractions > 50% with high cellular polymer content and yield, which were impossible with glucose that gave low polymer yields (<1 g/L) from 20 g/L under anaerobic conditions (Yamada et al., 2010). Gene disruptants (Δ*pta*, Δ*ackA*, Δ*poxB*, and Δ*dld*) that had been demonstrated to increase LA yield in *E. coli* (Zhou et al., 2011) were transformed with the set of genes for P(LA-*co*-3HB) production and cultivated on xylose (Nduko et al., 2014). The Δ*pta*, Δ*ackA*, and Δ*dld* mutants had higher polymer yields (6.5–7.4 g/L) compared with 6.3 g/L of the parent strain (*E. coli* BW25113). The Δ*pta* and Δ*dld* mutants also had higher LA fractions (58 and 66 mol%, respectively) in P(LA-*co*-3HB) compared with 56 mol% for the *E. coli* BW25113 (parent strain). When the Δ*pflA* and Δ*dld* mutations were combined, dual benefits were realized, i.e., high LA fraction (73 mol%) and high polymer yield (6.4 g/L from 20 g/L of xylose) with a polymer content of 96% (Nduko et al., 2014), underscoring the significance of metabolic engineering in modulating LA fractions in P(LA-*co*-3HB).

Another strategy that was used to exploit xylose utilization was the overexpression of the galactitol permease, GatC (Nduko et al., 2014), which is a non-ATP xylose transporter that was shown to transport xylose efficiently during LA production (Utrilla et al., 2012). This was intended to avoid the involvement of the main xylose transporter, xylE, a member of the ATP-binding cassette transporter encoded by the *xylFGH* operon, hence availing ATP for other cellular activities (Utrilla et al., 2012). *E. coli* (Δ*pflA*) harboring P(LA-*co*-3HB) production genes and overexpressing GatC produced 8.3 g/L of P(67 mol% LA-*co*-3HB) from 20 g/L xylose (polymer content 88%) compared with 7.7 g/L of P(58 mol% LA-*co*-3HB) and 79% polymer content of the parental strain expressing P(LA-*co*-3HB) production genes and GatC (Nduko et al., 2014). When xylose amount was doubled (40 g/L), the *E. coli* BW 25113 cells overexpressing GatC produced 14.4 g/L of P(66 mol%LA-*co*-3HB), the highest amount of polymer produced from pure sugars under the same culture conditions. Strains producing P(LA-*co*-3HB) having high LA fractions were found to have high LA in the medium. Production of 3HB is less efficient compared with LA. This is because to produce 3HB (C_4_) from pyruvate, two molecules of pyruvate (C_3_ × 2) are used with a loss of two carbon molecules as CO_2_, whereas two molecules of pyruvate produce two molecules of LA units (C_3_ × 2). This means that when P(LA-*co*-3HB) with high LA fraction is produced, copolymer yields will be high as well (Nduko et al., 2014). High-cell-density fed-batch fermentation using xylose and/or glucose as the carbon source was evaluated as a strategy to improve polymer production (Hori et al., 2019). In a glucose/xylose switching fermentation method, P(LA-*co*-3HB) concentrations of 26.7 g/L were attained. This study demonstrated a potential strategy of utilizing glucose and xylose mixture arising from hydrolysis of lignocellulosic biomass that overcomes carbon catabolite repression.

From these studies, a direct relationship between LA production in some strains and LA fraction in the P(LA-*co*-3HB) was found that indicated carbon redistribution with the mutant strains. In cases where there was incomplete utilization of xylose, LPE polymerization could have been the limiting step. Overexpression of GatC and fed-batch fermentation mode benefited polymer yields. These studies indicated that xylose could be a potent substrate for P(LA-*co*-3HB) production. As xylose is a major component of hemicellulose, its utilization could improve the economics of lignocellulose biomass use whereby only glucose is utilized efficiently. It was also demonstrated that fed-batch mode could be efficiently for P(LA-*co*-3HB) production from a mixture of glucose and xylose that could arise from lignocellulosic biomass hydrolysis.

### Lignocellulosic Biomass Hydrolyzates Use for P(LA-*co*-3HB) Production

As the production of P(LA-*co*-3HB) from the major lignocellulosic biomass sugars (pure glucose and xylose) had been demonstrated (Yamada et al., 2009; Nduko et al., 2014), efforts were made to produce P(LA-*co*-3HB) from biomass hydrolyzate. Sun et al. (2016) hydrolyzed *Miscanthus* × *giganteus* (hybrid *Miscanthus*) and rice straw using a commercial cellulase enzyme complex into a mixture of glucose and xylose. The hydrolyzates were used as a carbon substrate for P(LA-*co*-3HB) synthesis in *E. coli* BW25113 expressing LPE and 3HB-CoA and LA-CoA monomer supplying enzymes. The *E. coli* BW25113 growing on the *Miscanthus* × *giganteus* and rice straw hydrolyzates had a higher dry cell weight (9.2 and 9.4 g/L, respectively) than the same cells cultivated on a mixture of pure glucose and xylose (8.8 g/L). This was attributed to the presence of trace amounts of other sugars such as arabinose in the hydrolyzates. However, the polymer contents from the hydrolyzate cultures (56.9% and 55.9% for *Miscanthus* × *giganteus* and rice straw, respectively) and the mixture of the pure sugars (56.0%) were similar, whereas the LA fractions were 8.7 mol% for rice straw hydrolyzate, 16.9 mol% for *Miscanthus* × *giganteus* hydrolyzate, and 15.6 mol% for the glucose-xylose mixture. Glucose exerts carbon catabolite repression in the presence of other sugars, and this could happen in a hydrolyzate containing a mixture glucose and xylose (Ren et al., 2009). Kadoya et al. (2018) achieved simultaneous glucose and xylose utilization in *E. coli* by overexpressing a gene encoding Mlc, a multiple regulator for xylose and glucose uptake. By overexpressing Mlc, *E. coli* cells accumulated 13.6 g/L of P(LA-*co*-3HB) with polymer content of 65% and LA fraction of 11.8 mol%, which was higher compared with 4.8 g/L of P(LA-*co*-3HB) with polymer content of 50% and LA fraction of 14.4 mol% produced by the parental cells without Mlc overexpression (control) from a mixture of glucose and xylose. When the Mlc overexpression *E. coli* strain was cultivated on *Miscanthus* × *giganteus* hydrolyzate, it produced 10.6 g/L P(LA-*co*-3-HB) with polymer content and LA fraction of 58.6% and 10.9 mol%, respectively, which was also higher than 4.4 g/L of P(LA-*co*-3-HB) with polymer content of 47.3% and LA fraction of 9.1 mol% attained by the control cells (not expressing Mlc; Kadoya et al., 2018). These results exhibited the significance of Mlc overexpression in overcoming catabolite repression.

In another study, Takisawa et al. (2017) used eucalyptus hemicellulose hydrolyzate consisting mainly xylose and galactose for P(LA-*co*-3HB) production in *E. coli*. The dry cell weight attained was 8.6 g/L, polymer yield 5.4 g/L, polymer content 62.4%, and LA fraction of 5.5 mol%, which were comparable to those obtained from a mixture of pure xylose and galactose. However, the LA fraction in P(LA-*co*-3HB) from the hydrolyzate was lower than that obtained from the pure sugars (16.9 mol%). The low LA fraction in P(LA-*co*-3HB) obtained from the hemicellulose hydrolyzate was attributed to catabolite repression in *E. coli*, although the results demonstrated the potential of eucalyptus hemicellulose hydrolyzate for P(LA-*co*-3HB) production.

During lignocellulose biomass pretreatment, some toxic byproducts such as furfural, hydroxymethylfurfural, and acetate are produced (de Paula et al., 2019). These compounds can affect microbial growth and affect polymer yield when the hydrolyzates are used as substrates for polymer production (Nduko et al., 2012b). As a result, a number of processes have been recruited to detoxify the hydrolyzates before use in fermentation. However, these detoxifying steps add to the overall cost of polymer production. These have triggered the search for resistant organisms. For instance, Salamanca-Cardona et al. (2017) identified *E. coli* strains that were tolerant to acetate, whereas Nduko et al. (2012b) identified *E. coli* LS5215 to be resistant to 5-hydroxymethylfurfural. These strains could thus be useful in the production of P(LA-*co*-3HB) from lignocellulosic biomass hydrolyzates.

**FIGURE 5:**
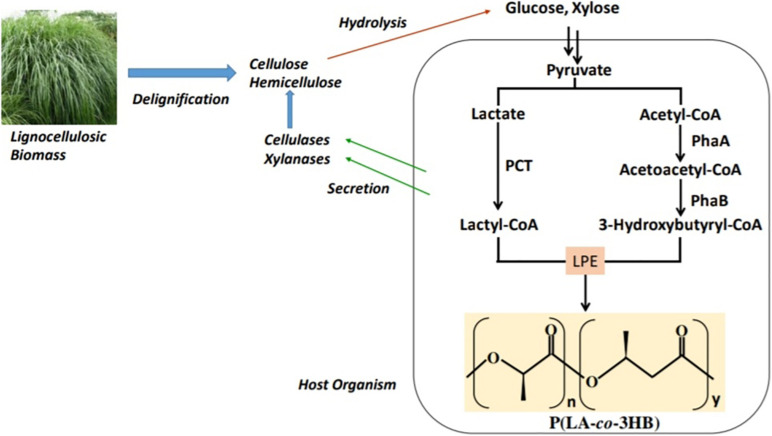
A consolidated bioprocess where the host is engineered to combine cellulose and/or hemicellulose hydrolysis and P(LA-*co*-3HB) production. PCT, propionyl-CoA trabnsferase; PhaA, β-ketothiolase; PhaB, NADPH-dependent acetoacetyl-CoA reductase; and LPE, lactate (LA)–polymerizing enzyme.

The two-step system of hydrolysis followed by fermentation for the production of P(LA-*co*-3HB) could be costly because hydrolysis is done at high temperatures and low pH using acids or high pH using alkaline compounds. Therefore, a process that consolidates hydrolysis of biomass and fermentation for the production of P(LA-*co*-3HB) could be cost-friendly (Figure 5). *E. coli* that has been engineered for P(LA-*co*-3HB) lacks xylan hydrolytic enzymes (endoxylanases and β-xylosidases; Subramaniyan and Prema, 2002). To obtain a xylanolytic *E. coli*, Salamanca-Cardona et al. (2014a) expressed an endoxylanase (XylB) and a β-xylosidase (XynB) in *E. coli* LS5218 engineered for P(LA-*co*-3HB) production. The resultant strain produced 3.7 g/L P(LA-*co*-3HB) from a media containing xylan supplemented with xylose, hence the first consolidated bioprocess (CBP) for P(LA-*co*-3HB) production from xylan. However, this CBP attained low (<4 mol%) LA units in P(LA-*co*-3HB). To increase the LA ratio in this system, Δ*pflA* mutation was introduced in xylanolytic *E. coli* LS5218 expressing P(LA-*co*-3HB) biosynthesis enzymes and the cells cultivated on a media containing beechwood xylan (Salamanca-Cardona et al., 2014b). The inactivation of *pflA* resulted in the production of P(LA-*co*-3HB) with enhanced LA fraction (up to 18.5 mol%) in the copolymer from xylan or xylan supplemented with xylose, underscoring the essentiality of high LA production on the LA incorporation into P(LA-*co*-3HB).

The CBP by Salamanca-Cardona et al. (2014a) was a quasi-CBP that required xylose supplementation, and for a strict CBP system to be established, Salamanca-Cardona et al. (2016) established a xylanolytic plasmid system in *E. coli* JW0885 (Δ*pflA*) expressing P(LA-*co*-3HB) biosynthesis enzymes. The xylanolytic *E. coli* JW0885 produced P(LA-*co*-3HB) containing 19.2 mol% LA. This system can, however, be optimized to establish a strict CBP system that could be used for P(LA-*co*-3HB) production from xylan, potentially contributing to the reduction of the cost of producing P(LA-*co*-3HB).

## Properties of LA-Based Polymers

### Enantiomeric Purity of LA-Based Polymers

Enantiopurity of polymers is a significant factor that affects thermal properties of PLA (Worch et al., 2019). Commercial PLA is made either from L- or D-LA, resulting in P[(*S*)-LA] (PLLA) and P[(*R*)-LA] (PDLA) homopolymers, which are highly crystalline (Masutani and Kimura, 2014). Crystalline homopolymers have high stability near their melting points (*T*_m_). L-Lactide generates a semicrystalline PLA with a melting transition of around 180°C and glass transition temperature (*T*_g_) of approximately 67°C, whereas an amorphous stereocomplex of 1:1 L- or D-lactides has a *T*_g_ near room temperature and a *T*_m_ of 220–230°C. Enantiopurity thus affects polymer properties. P(LA-*co*-3HB) was found to have (*R*)-LA and (*R*)-3HB (Taguchi et al., 2008), which could be attributed to the enantiospecificity of PHA synthases, i.e., are specific to *R*-form monomers (Muhammadi et al., 2015) that were inherited by LPE. With chemical synthesis of P(LA-*co*-3HB) not attaining 100%, i.e., the biosynthetic system enables the synthesis of optically pure [P(LA-*co*-3HB)].

### Sequential Structure and Molecular Weight of LA-Based Polymers

Polymer structure, especially monomer sequence, affects the properties of a polymer (Wolf et al., 2009; Lutz et al., 2013; Matsumoto et al., 2018a). High-resolution NMR is an effective method for determining structural and monomer sequence of copolymers (Kricheldorf, 2001). Sequence information could be obtained by ^1^H NMR. Analysis of P(LA-*co*-3HB) detected LA-LA-LA, LA-LA-3HB, 3HB-LA-LA, and 3HB-LA-3HB triads, whereas ^1^H–^13^C COSY-NMR profiles detected LA-LA-LA triads and LA-LA dyads. This revealed sequential polymerization of LA units by LPE. All the triad sequences were detected in P(LA-*co*-3HB) that suggested random distribution of LA and 3HB monomer units in the copolymer (Yamada et al., 2009). Molecular weight of a polymer affects its tensile strength (Kricheldorf, 2001). In analysis of P(LA-*co*-3HB) polymers, it was found that molecular weight (M_w_) of P(LA-*co*-3HB) was dependent on the LA/3HB ratios; i.e., there was an inverse relationship between LA fraction and M_w_ (Shozui et al., 2010b; Yamada et al., 2011; Song et al., 2012; Salamanca-Cardona et al., 2017; Nduko and Taguchi, 2019). This relationship was also maintained with the thermal properties (*Tg* and *Tc*) of the copolymers.

### Thermal and Mechanical Properties of LA-Based Polymers

The thermal properties of PHAs determine their ideal processing and utilization temperatures and affect polymer quality in terms of parameters such as crystallization and heat resistance. PLA homopolymer is either amorphous or semicrystalline (40–60% crystallinity; Mehta et al., 2005). For amorphous PLA, its *T*_g_ is the determinant of its upper use temperature, whereas in semicrystalline PLAs, both *T*_g_ (approximately 58°C) and *T*_m_ (130–230°C) determines the use temperatures of the polymer. The *T*_g_ and *T*_m_ are both dependent on the molecular weight, thermal history, optical composition, and the primary structure of the polymer. PHB is semicrystalline with a *T*_m_ of 174–179°C and *T*_g_ of −7°C (Anjum et al., 2016). PLA is a transparent polymer whereas P(3HB) is an opaque polymer, which is consistent with their *T*_g_ (Nduko et al., 2015). Microbial P(LA-*co*-3HB) exhibited intermediate Tg (−6 to 34°C), which varied with the LA/3HB ratio (Yamada et al., 2011). The *T*_m_ and melting enthalpy (Δ*H*_m_) of P(LA-*co*-3HB) with 29–47 mol% LA were lower than those of P(3HB) and PLA homopolymers. The lower *T*_m_ and Δ*H*_m_ of P(LA-*co*-3HB) could be attributed to low crystallinity due to copolymerization as compared with the respective homopolymers. Copolymerization could allow the customization of thermal properties and widens the narrow processing window of PLA and PHB homopolymers.

Solid materials made from high M_w_ (>100 kDa) PLA are strong, whereas those made of PLA of low M_w_ (<70 kDa) are weak and ductile (Moon et al., 2001a,b; Cicero et al., 2002; Södergård and Stolt, 2002; Castro-Aguirre et al., 2016). The increase in strength with *M*_*w*_ is, however, accompanied with decrease in elongation-at-break (Engelberg and Kohn, 1991). On its part, P(3HB) is brittle at low Mw; for instance, a 22-kDa polymer elongates only 2% at break with a force of 8.5 MPa (Srubar et al., 2012). P(3HB)s strength improves with increase in Mw. For example, at 370 kDa, strength is 36 MPa, with some alterations in strain behavior (Engelberg and Kohn, 1991; Cox, 1994), whereas at 11 MDa, P(3HB)’s strength is 62 MPa, and elongation-at-break is 58% (Kusaka et al., 1999). Assessment of microbial P(LA-*co*-3HB) found its Young modulus to be inversely related to the LA fraction in the copolymer and ranged between 148 and 905 MPa, which was lower than that of PLA (1,020 MPa), and P(3HB; 1,079 MPa) homopolymers (Yamada et al., 2011). P(LA-*co*-3HB) were more flexible compared with PLA and P(3HB) homopolymers. For instance, the elongation at break of the P(29 mol% LA-*co*-3HB) was 156% (Yamada et al., 2011). This indicated that P(LA-*co*-3HB) is more elastic than PLA and P(3HB) homopolymers; hence, the alteration of LA fractions in P(LA-*co*-3HB) could lead to the formation of materials with desired mechanical properties.

## Microbial Degradation of P(LA-*Co*-3HB)

Polyhydroxyalkanoates, in particular P(LA-*co*-3HB), are promising materials with potential practical applications. With perceived increase in their production, efforts should be made to study P(LA-*co*-3HB) degradation in the environment. PHA degradation generates products (CO_2_, H_2_O, methane, and LA) that are safe to the environment (Volova et al., 2017). In nature, P(3HB) is degraded by microbes expressing extracellular PHA-depolymerases, whereas PDLA is not easily biodegraded (Kawai et al., 2011). The biodegradation of P(LA-*co*-3HB) will be dependent on the degradability LA and 3HB components in the copolymer. Sun et al. (2014), screened soil samples from Sapporo, Japan, for microorganisms degrading P(LA-*co*-3HB). They used P(67 mol% LA-*co*-3HB) as the substrate and isolated a microorganism identified as *Variovorax* spp. C34 that had high P(LA-*co*-3HB) and P(3HB) degradation ability but was not degrading PDLA. Phylogenetic analysis using 16S rRNA gene sequencing found *Variovorax* spp. to be close to the members of the *Comamonadaceae* family such as *Acidovorax* sp. TP4 (Jendrossek et al., 1993), *Comamonas* sp. (Kasuya et al., 1994), *Comamonas testosteroni* (Kusaka et al., 1999), and *Delftia acidovorans* (Kasuya et al., 1997) that secrete extracellular P(3HB) depolymerases. In the supernatant of the *Variovorax* spp. C34 culture, P(LA-*co*-3HB) degradation was detected, indicating that the organism produced an extracellular depolymerase(s). The P(LA-*co*-3HB) depolymerase was purified and found to degrade P(LA-*co*-3HB) and P(3HB), but not PLLA or PDLA (Sun et al., 2014). The degradation of P(LA-*co*-3HB) was faster than the degradation of P(3HB), and this could be attributed the lower crystallinity in P(LA-*co*-3HB) compared with the higher crystallinity of P(3HB).

In another study, Hori et al. (2020) screened for bacterial groups degrading P(D-LA-*co*-3HB) from soil. Specific emphasis was on the degradation of D-LA clustering units in the copolymer using chemically synthesized D-LA homo-oligomers. Based on clear zone formation on agar plates containing P(64 mol% D-LA-*co*-3HB) and 16S rRNA sequence analysis, eight *Variovorax*, three *Acidovorax*, and one *Burkholderia* strains were isolated and these strains were closely related to previously identified PHA-degrading bacteria (Sun et al., 2014). The isolated strains were found to consume P(D-LA-*co*-3HB) in a liquid culture, although a D-LA fraction remained unconsumed, and this was attributed to a D-LA clustering structure in the copolymer. When the isolated strains were cultivated with the D-LA homo-oligomers, it was found that degradation was dependent on the degree of polymerization (DP). For instance, six *Variovorax* and one *Acidovorax* strains partly consumed oligomers with 10–30 DP. On the other hand, oligomers with DP of 20–60 were not consumed by the isolates, indicating DP as the degradation determining factor. With molecular dynamic simulation, it was proposed that the upper cutoff DP for D-LA homo-oligomer degradation could be determined by conformational structure of the oligomers in water, and this is particularly important in molecular design of biodegradable D-LA–containing polymers.

## Production of LA Oligomers

Since Taguchi et al. (2008) reported for the first time the microbial factory for the synthesis of P(LA-*co*-3HB), a number of attempts have been made to enhance the LA fraction in the polymer (Yamada et al., 2009, 2011; Song et al., 2012; Nduko et al., 2013a, 2014). In these studies, an unusual phenomenon regarding the molecular weights of the polymers has been observed. The molecular weights (M_n_ and M_w_) of PLA-like polymers and PLA were lower (magnitude of 10^3^) than those of P(LA-*co*-3HB) with <70 mol% LA fraction (10^4^–10^5^; Shozui et al., 2011; Song et al., 2012; Nduko and Taguchi, 2019). The low molecular weights with high LA fractions in P(LA-*co*-3HB) suggested the possible production of LA oligomers by the recombinant microorganisms. These oligomers, if produced, can be purified and chemically catalyzed in a short-cut process to generate PLA.

**FIGURE 6:**
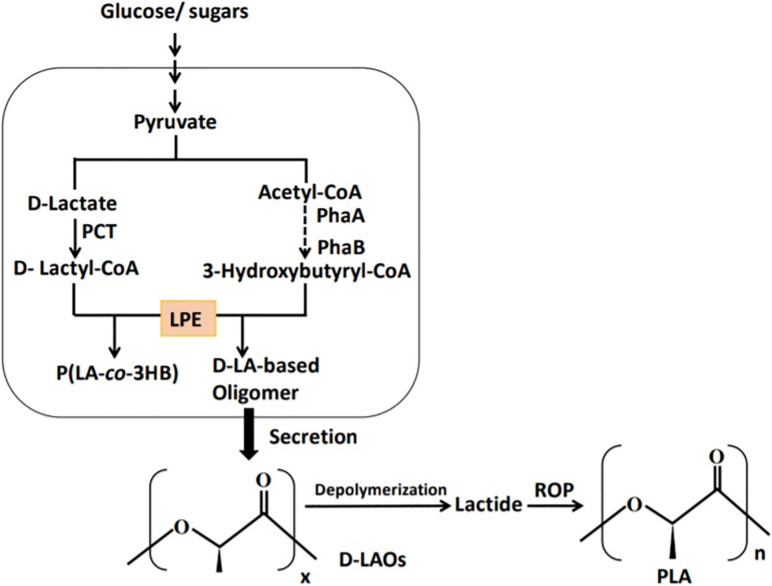
Schematic presentation of the P(LA-*co*-3HB) production system adapted for the production of D-LA–based oligomers. PCT, propionyl-CoA trabnsferase; PhaA, β-ketothiolase; PhaB, NADPH-dependent acetoacetyl-CoA reductase; and LPE, lactate (LA)–polymerizing enzyme.

The possibility of LA oligomer production in the P(LA-*co*-3HB) biosynthesis system was explored by Utsunomia et al. (2017c) as earlier discussed by Nduko and Taguchi (2019). *E. coli* expressing LPE, PCT, PhaA, and PhaB (Taguchi et al., 2008) was cultivated on glucose and the supernatant examined by NMR (Figure 6). The chemical shifts (δ in ppm) in ^1^H NMR of the extracted fraction of the supernatant were similar to those of P(LA-*co*-3HB), indicating the presence of LA oligomers in the supernatant. The slight differences between supernatant extract and P(LA-*co*-3HB) polymer was linked to the molecular weight differences between the polymers and oligomers (Yamada et al., 2009; Nduko et al., 2012a; Utsunomia et al., 2017c). Based on ^1^H NMR, the oligomers in the supernatant had 63 mol% LA fraction (Utsunomia et al., 2017c). The electrospray ionization time-of-flight mass spectrometry (ESI-TOF-MS) examination of the extract found a bimodal distribution of the oligomers with periodic m/z values of between 400 and 1,400, which indicated that the oligomers were between ∼4- and 19-mer comprising both LA and 3HB units. The peak tops of the bimodal distribution were 7- and 12-mer, for LA and 3HB, respectively.

The researchers sought to enhance LA oligomer secretion from P(LA-*co*-3HB) polymer–producing *E. coli* by the inclusion of chain transfer (CT) agents in the culture media. In the presence of CT agents, PHA synthase will lose polymerization activity and instead transfer polymer chain to a CT agent containing a hydroxyl group (Madden et al., 1999; Miyahara et al., 2019). This will lead to synthesis of polymers with low molecular weight and enhance the production of oligomers (Hiroe et al., 2013). Some of the CT agents that have been demonstrated to reduce molecular weight of PHAs include polyethylene glycols and short-chain alcohols (Shi et al., 1996; Ashby et al., 1997; Tomizawa et al., 2010; Hiroe et al., 2013; Thomson et al., 2014). Utsunomia et al. (2017c) evaluated four CT agents for their effectiveness in enhancing LA oligomer secretion. It was found that diethylene glycol (DEG) was the most effective in enhancing LA oligomer production, concomitantly reducing molecular weight of P(LA-*co*-3HB) in the cells.

The enhanced LA oligomer secretion into the medium with the addition of DEG was not as a result of cell lysis (Utsunomia et al., 2017c). With 5% DEG addition into the medium, LA oligomer yield of 8.3 ± 1.5 *g* L^–1^ was obtained with LA fraction of 86.0 ± 4.5 mol%. Upon extraction from the supernatant, 3.2 *g* L^–1^ of the LA oligomers was recovered and was free of DEG, although ^1^H-^1^H COSY-NMR indicated that LA oligomers were covalently capped with DEG at their carboxy terminal (Utsunomia et al., 2017c). Similarly, ^1^H-^1^H COSY-NMR and ^1^H-^1^H DOSY NMR revealed that the high-molecular-weight P(LA-*co*-3HB) accumulated in the cells was also terminated with DEG at the carboxyl terminal. The ESI-TOF-MS analysis of LA oligomers produced with addition of DEG detected periodic m/z values in the range 400–800 that corresponds to ∼4- to 10-mers, suggesting shorter oligomers were generated with addition of DEG compared with those without (Utsunomia et al., 2017c).

Evaluation of LA concentration found that intracellular LA oligomer (1.8 g/L) in *E. coli* cells was higher than extracellular concentration (0.4 g/L), pointing out to the presence of a secretion barrier (Utsunomia et al., 2017c). To pinpoint the secretion route in *E. coli* for LA oligomers, a loss-of-function screening strategy was adopted. This involved screening of 209 deletants of membrane proteins known to transport organic compounds (Utsunomia et al., 2017a). Cultivation of the deletants found that 55 of them had reduced extracellular LA oligomer concentration compared with the parental strain. There was interest in mutants with elevated intracellular LA oligomers as this could indicate disruption of LA secretion mechanism. Consequently, seven mutants (Δ*mpF*, Δ*argT*, Δ*mngA*, Δ*macA*, Δ*ompG*, Δ*citA*, and Δ*cpxA*) exhibiting either decreased or increased LA oligomer secretion were cultivated without plasmids and were found to grow normally. This suggested that the membrane proteins that were deleted were not injurious to cell growth. On the basis of these results, it was proposed that secretion of LA oligomers through the outer membrane was mediated by passive diffusion via porins including OmpG and OmpF. To pass through the inner membrane, inner membrane–associated proteins such as ArgT, CitA, CpxA, and MacA were proposed to be transporting LA oligomers (Utsunomia et al., 2017a).

To demonstrate the applicability of the LA oligomers for PLA synthesis, lactide was synthesized from LA oligomers (68 mol% LA, 3- to 7-mers) produced by *E. coli* growing on glucose with 5% DEG (Utsunomia et al., 2017b). Alongside this, LA oligomers with 61 mol% LA with 3- to 16-mers synthesized by *E. coli* cultivated on glucose supplemented with L-LA homo oligomers (100 mol% LA) and mers ranging from 3 to 14 were used as a control. In either case, zinc oxide was used as a catalyst to generate the lactides. Although D-LA oligomers could form lactides, their conversion rates (25% for D-LA oligomer and 13% for D-LA oligomer-DEG) were lower when compared to 77% obtained with the control (L-LA oligomers; Utsunomia et al., 2017b). The lower conversion rate of D-LA oligomers was attributed to the presence of 3HB units that could hinder lactide conversion rates. As a result, enhancing the LA fraction in the D-LA oligomers might increase the conversion rate. In subsequent experiments, xylose was used to produce LA oligomers having 97 mol% LA. On extraction from the supernatant, LA oligomers containing 89 mol% LA were obtained, and when used for lactide synthesis, the conversion rate was improved (Utsunomia et al., 2017b). This indicated that use of LA oligomers with high LA fraction is essential for improved lactide conversion. In addition, molecular weight of the oligomers could affect lactide yield as short oligomers vaporize and get lost during heating. Therefore, production of longer oligomers is a potent research area to increase lactide synthesis.

## Conclusion and Future Outlook

Polylactic acid and PHAs have attracted much attention in recent decades in research as well in industry. These materials have versatile properties; however, the major drawback of these polymers is their production cost. For the production of high-molecular-weight PLA, ROP has found industrial use. Coupling ROP with advances in fermentative production of LA has reduced PLA production cost. Despite the advances in ROP chemistry and life-cycle assessments of PLA production compared to equivalent fossil fuel–derived plastics being positive, the multistep chemo–bio process for PLA production is still considered to be complex and expensive (Matsumoto and Taguchi, 2010; Lasprilla et al., 2012; Vink and Davies, 2015; De Clercq et al., 2018). Lactide formation from LA production involves many purification steps (De Clercq et al., 2018), which contribute approximately 30% of the cost of PLA, hampering its competitiveness against fossil fuel–derived plastics (Corma et al., 2007; Van Wouwe et al., 2016).

Consequently, researchers have undertaken crucial steps to improve PLA synthesis and make it more feasible. One such strategy is the establishment of a single-pot bioprocess for PLA synthesis analogous to the synthesis of PHAs. This was, however, achieved in 2008 (Taguchi et al., 2008), where a PHA synthase was engineered to acquire LA-polymerizing activity. The engineered PHA synthase termed LPE was coupled with LA-CoA supplying enzyme (PCT) and 3HB monomer supplying enzymes to establish a whole-cell bioprocess for the production of LA-based polymers, P(LA-*co*-3HB). Analysis of the P(LA-*co*-3HB) found that material properties varied with the LA fraction in the copolymer. As the pioneering study for P(LA-*co*-3HB) achieved only low LA fractions in the copolymer, enzyme evolutionary engineering, metabolic engineering, host change, disruption of σ factors, and fermentation manipulation techniques (Table 1) were recruited to improve the LA fractions in P(LA-*co*-3HB) and eventual production of PLA in microorganisms. The PLA synthesized by microorganisms had similar properties to the chemically synthesized PLA.

The price of the substrate used as the carbon source for PLA and P(LA-*co*-3HB), polymer yield on the carbon source, and downstream processes determines the ultimate cost of the polymers. Lignocellulosic biomass is abundant in nature and offers an inexpensive carbon source for the production of PLA and LA-based polymers. As has been demonstrated, glucose and xylose, the major components of cellulose and hemicellulose, respectively, are good substrates for the production of LA-based polymers with high LA fractions. Furthermore, lignocellulose biomass hydrolyzates and xylan have been shown as substrates for production of LA-based polymers. If more effort is directed toward optimizing the processes and utilizing all biomass components, then the cost of producing LA-based polymers and PLA will reduce and make the polymers competitive.

The microbial synthesis of high LA P(LA-*co*-3HB) and PLA suffered in terms of polymer yields and molecular weight. At LA fractions in P(LA-*co*-3HB) greater than 70 mol% and in PLA biosynthesis, only small amounts of polymer are synthesized, and the polymers are of low molecular weight (Matsumoto et al., 2018b). It was hypothesized that rather than substrate specificity of LPE, polymer size limitation was due to the molecular dynamics of PDLA. Molecular dynamics of the polymer could be affected by the Tg of the synthesized polymer. High-molecular-weight PLA has a *T*_g_ of approximately 60°C, which reduces with decrease in M_w_. As cultivation temperatures for polymer production was 30°C, the *T*_g_ of low M_w_ PLA like polymers and PLA could have fallen below this, gradually stalling polymer synthesis. Thus, efforts to overcome this stalling mechanism need to be undertaken to improve polymer yield and molecular weight. During degradation experiments of P(LA-*co*-3HB) and D-LA oligomers, it was found that the DP affected degradation of D-LA oligomer, whereby long D-LA oligomers are not degraded. Therefore, efforts are needed on the molecular design of biodegradable D-LA–containing polymers.

The P(LA-*co*-3HB) biosynthesis system was found to secrete LA oligomers that were converted into lactides, antecedents for chemical PLA production. Endeavors have been made to unravel LA-oligomer secretion mechanism. However, examinations to pinpoint the precise mechanism to better understand candidate transporters are vital as this will direct targeted plans to advance LA oligomer secretion. Despite the fact that the produced oligomers were forming lactides mediated by a catalyst, the conversion rate was low, a phenomenon attributed to the presence of 3HB units in the chain. Therefore, efforts should be directed toward the PLA synthesis system to generate LA oligomers that could be coupled with the chemical step for the synthesis of high-molecular-weight PLA. In addition, improving the oligomer yields is another research frontier. Strategies to increase the yield of PLA, P(LA-*co*-3HB), and LA oligomers along with the utilization of lignocellulosic biomass and other agroindustrial byproducts have potential of creating a sustainable biorefinery for the production of LA-based materials.

The systems discussed herein have scale-up potential and could improve the competitiveness of LA-based polymers and materials against the petrochemical-derived counterparts. Along with exploration of substrates for production, polymer yields, monomer composition and sequence, and oligomer production, the scale-up potential should be evaluated. As P(LA-*co*-3HB) are biodegradable, they could emerge as ecofriendly alternatives to the petrochemical-based plastics.

## Author Contributions

ST conceived and presented the idea. JN wrote the manuscript with the support of ST. ST and JN discussed and contributed to the final version of the manuscript. Both authors contributed to the article and approved the submitted version.

## Conflict of Interest

The authors declare that the research was conducted in the absence of any commercial or financial relationships that could be construed as a potential conflict of interest.
